# Development and validation of conflict management attitude questionnaire for medical students

**DOI:** 10.1186/s12909-022-03928-0

**Published:** 2022-12-12

**Authors:** Fatemeh Mohseni, Aeen Mohammadi, Mahboobeh Khabaz Mafinejad, Larry D. Gruppen, Nasim Khajavirad

**Affiliations:** 1 Department of Medical Education, Tehran University of Medical Sciences, Tehran, Iran; 2 Education Development Center, Gerash University of Medical Sciences, Gerash, Iran; 3 Department of E-Learning in Medical Education, School of Medicine, Tehran University of Medical Sciences, Tehran, Iran; 4 Health Professions Education Research Center, Education Development Center, Department of Medical Education, Tehran University of Medical Sciences, Tehran, Iran; 5 PhD, Department of Learning Health Sciences, University of Michigan, Ann Arbor, MI USA; 6 Department of Internal Medicine, Imam Khomeini Hospital Complex, Tehran University of Medical Sciences, Tehran, Iran

**Keywords:** Conflict management, Psychometric, Medical students, Development of questionnaire

## Abstract

**Background:**

Medical students should effectively manage conflicts in teamwork and communication with other team members. This study aimed to develop and validate a tool to evaluate attitude of medical students and physicians toward conflict management.

**Method:**

A multi-step process was employed to develop and validate a Conflict Management Attitude Questionnaire (CMAQ) based on the steps recommended in AMEE Guide No. 87. First, the initial items were obtained from the literature review and focus group. After cognitive interviews with the medical students and revision of the questionnaire, content validity was performed by experts. The construct validity and reliability of the questionnaire were assessed using exploratory Factor Analysis (EFA) and Cronbach’s alpha coefficient, respectively.

**Results:**

This multi-step process resulted in a 12-item, five-point Likert-type questionnaire with satisfactory construct validity. Exploratory factor analysis revealed three factors, comprising the four items from the "perceived interactions in conflict management" subscale loading on the first factor, and five items from the "perceived value of learning conflict management" subscale loading on the second factor, along with three items from the "perceived application of conflict management" subscale loading on the third factor. All subscales described 56.48% of the variance. Validation results showed that Content Validity Index (CVI) and Content Validity Ratio (CVR) were greater than 0.75. Cronbach's alpha coefficient was 0.791.

**Conclusion:**

This study showed that CMAQ has valid evidence for assessing the attitude of medical students toward conflict management with favorable psychometric properties and strong evidence of construct validity. However, due to the lack of evidence on any specific questionnaire to evaluate the attitude towards conflict management, future studies should conduct a confirmatory investigation regarding other aspects of medical students' attitudes toward conflict management.

**Supplementary information:**

The online version contains supplementary material available at 10.1186/s12909-022-03928-0.

## Introduction

Conflict arises as a result of the lack of coordination or incompatibility among individuals due to differences in their needs, beliefs, attitudes, values, goals, ideas, or interests [1, 2]. Interpersonal and team conflicts are the most common types of conflict experienced by healthcare team members [3]. Conflicts in healthcare teams, where individuals work together on a specific task, are the most significant obstacles to deliver effective service [3, 4]. Evidence shows that conflict in healthcare team is inevitable, which is not necessarily considered a negative aspect. Effective conflict management can lead to more effective participation of the team members and group dynamics, improved patient care, job satisfaction, and professional performance [5, 6]. Moreover, poor conflict management can lead to difficulties in providing healthcare services and additional financial burdens on the education and healthcare system [7, 8].

In recent decades, the quality of healthcare service provision and patient safety have been considered two core indicators in healthcare system [9]. According to previous studies, the cooperation of healthcare team members and appropriate management of conflicts play a vital role in providing high-quality and safe care services [10]. Inappropriate consequences of conflicts mismanagement on healthcare provision quality have caused professional societies and accreditation institutions to demand the implementation of conflict management training programs to teach health literacy [7, 11, 12].

Evidence frequently showed that physicians, who work the front line of healthcare teams, most expectedly encounter conflicts [13–15]. Therefore, the capability of managing conflict as a core competency for physicians and leaders of the healthcare team is vital [3, 16]. Although medical students frequently encounter conflicts in their professional lives, the education system does not prepare them to acquire conflict management capability and fortify it [17]; for this capability, formal teaching, required to improve physicians’ teamwork performance, has been deficient [3].

Establishing appropriate behavior and teaching conflict management competency starting early in one’s education path is considerably more effective than changing wrong behaviors and beliefs institutionalized in individuals [6]. According to previous studies, attitudinal barriers may also affect conflict management capability. Improving attitude can affect student performance in conflict management [18–21]. Evidence emphasizes the positive effect of education on the attitudes of medical students and physicians [22, 23].

Exacerbating these concerns is a lack of an effective tool for evaluating attitudes toward conflict management among medical students in undergraduate medical curricula. Although only a limited number of studies have examined students’ attitudes toward conflict management [24–28], one study has addressed the possibility of assessment of students’ attitudes towards Interprofessional Education (IPE) after participating in a conflict management training course [29].

To the best of our knowledge, no specific tool for evaluating the conflict management of medical students is available. There is a need for developing such a questionnaire that would be beneficial for teachers and educational planners alike, which would also allow for an evaluation of the effect of training courses. Therefore, this study aimed to develop and validate a questionnaire to evaluate the attitudes of medical students and physicians toward conflict management.

## Methods

### Context

The undergraduate medical curriculum at Tehran University of Medical Sciences (TUMS) is a prestigious and high-rank course among undergraduate medical education programs in Iran. Medical students with a high rank in the centralized national entrance exam are accepted. This program includes four phases; basic science (two years), pathophysiology (one year), clerkship (two and half years), and internship (one and half years).

### Design

**Fig. 1:**
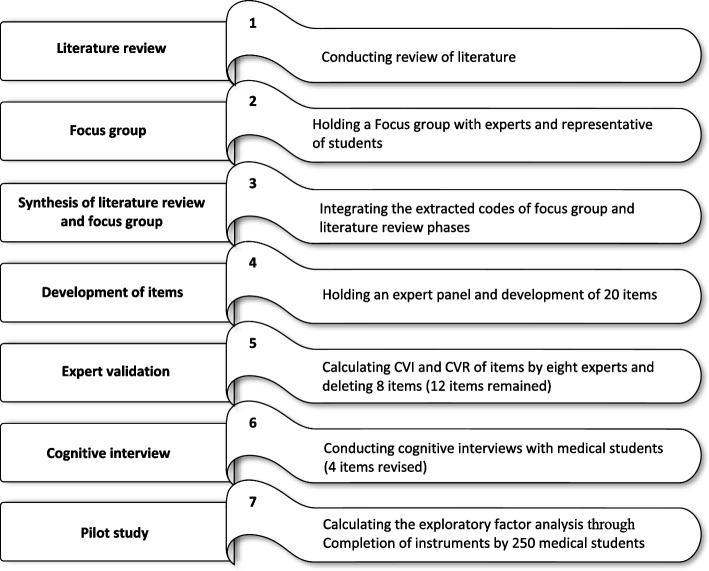
The steps of instrument development and validation

Conflict Management Attitude Questionnaire (CMAQ) based on the seven stages recommended by Artino et al. in AMEE Guide No. 87 (Fig. 1) was designed [30], a psychometric study to evaluate CMAQ validity and reliability.

## Questionnaire development

### Step 1: literature review

A systematic search was conducted on several databases (e.g., ProQuest, ERIC, Scopus, WOS, PubMed, and Google Scholar search engine) focusing on the tools assessing medical students’ attitude towards conflict management in literature until January 9, 2021 through the following keywords: "conflict", "manag*", "resolution", "instrument", "tool", "questionnaire", "Survey", "students, medical", "medical student*", "Education, Medical", "medical education", "residen*" and "medical trainees". The search strategy for one database (PubMed) is presented in Appendix 1. The first researcher (FM) analyzed full texts using content analysis method. First, FM has read through the full text to obtain a sense of the whole and familiarize with the data. The analysis process was conducted by determining the meaning units relevant to the research aim. Each identified meaning unit was labeled with a code to enable abstraction. Then, the extracted codes were checked in two sessions with the attendance of the second researcher (MKM). In case of disparity, the agreement was reached via consensus.

### Step 2: focus group

The focus group session followed the Association for Medical Education in Europe (AMEE) guideline on using focus groups [31]. We conducted a 2-h focus group with eight participants consist four conflict management experts and four medical students. We also recruited participants through the purposive sampling method. The inclusion criteria were: 1) experts had more than two years of experience in teaching and assessing conflict management and 2) medical students who attended more than one year in the clinical phase and experienced a conflict situation at least once. During the session, the coordinator asked the participants to explain their viewpoints, feelings, and beliefs regarding conflict management through directed questions (e.g., what matters are involved in conflict management? How did you respond to this conflict?). The moderator emphasized that there were no correct or incorrect answers. The meeting was audio recorded with the consent of the participants and then transcribed verbatim. Two researchers (FM and MKM) jointly classified the codes through content analysis.

### Step 3: Synthesis of the literature review and focus group

The codes extracted through the focus group were integrated with the codes identified through a literature review by two researchers (FM and MKM). They repeatedly compared the combined codes with the initial codes to ensure comprehensiveness. After that, codes with common and similar concepts were preserved, and duplicate codes were eliminated.

### Step 4: development of items

Six experts in the field of medical education and communication skills were invited as expert panel members to guide the development of CMAQ items. A two-hour session was held on an online platform. The meeting coordinator presented the resulting codes, and then comments were received to write the most appropriate statements for CMAQ items.

### Step 5: expert validation

In order to evaluate content validity, eight experts in the field of medical education and communication skills were selected. They were explained the objectives of the study and the procedure for obtaining their opinions on the content validity assessment process. Then, the CMAQ was emailed to them along with the content validity assessment form, and the Content Validity Index (CVI) and Content Validity Ratio (CVR) were calculated. To calculate the CVI index, experts were asked to rate the relevance and clarity of each item on a four-point Likert scale ranging from 1 (not relevant) to 4 (very relevant). The CVI index was calculated using the formula developed by Waltz and Bausell [32]. If the value was above 0.79, the CVI index was confirmed [32]. The Lawshe formula [33] was used to calculate the CVR index. Experts were asked to rate each item on a three-point Likert scale (necessary; useful but not important; not necessary). Items that had a CVR of over 0.75 were retained [33].

### Step 6: cognitive interview

Face validity was examined by cognitive interviews. In this study, cognitive interviews were conducted with four medical students at TUMS. In each interview, the students were asked to read each item aloud and concurrent verbal probing recount their understanding of its meaning in their own language. Students’ understanding of each item was checked after reading it, and the questions and statements related to each item were verified. Perceived biases and word ambiguities were corrected. Items that assessed as similar from the same domain were removed, and confusing or inappropriate items were revised.

### Step 7: pilot testing

To assess construct validity and reliability, the CMAQ was designed as a Google form and its link was sent to social media applications (WhatsApp). Exploratory Factor Analysis (EFA) was performed to determine the underlying factors of conflict management [34]. To determine factor loadings, the questionnaire was completed by a random sample of 250 medical students. The data were analysed using IBM SPSS Statistics version 26 [35]. Principal component analysis with Varimax rotation was used to perform EFA. The KMO index was used to evaluate the adequacy of the sample size (greater than 0.7) and Bartlett's test was used to ensure the suitability of the factor analysis (*p*-value less than 0.05). In the final version, the items with a factor loading of more than 0.40 were considered suitable. After conducting the EFA and identifying the factors, Cronbach's alpha was calculated to assess internal consistency. A Cronbach's alpha of more than 0.7 was considered acceptable [36].

## Results

The initial draft of CMAQ included 20 items on a Likert scale (strongly disagree, disagree, neither agree or disagree, agree, and strongly agree). Examining the CVI index showed that all CMAQ items had scores higher than 0.79 in terms of relevance and clarity; only one item had a score less than 0.75, which was removed from the questionnaire. However, regarding the CVR index, eight items 1, 3, 5, 13, 15, 17, 18 and 19 scored less than 0.75 and were removed from the questionnaire. At this stage, 12 items remained in the CMAQ draft. Table 1 presents the results of the tool’s CVI and CVR indicators. After the cognitive interview, four items that considered difficult to understand by the students were reviewed by the research team (Table 1).

**Table 1:** CVI and CVR index values

Item	CVI		Item-CVI	CVR	
	**Relevance**	**Clarity**			
**1**	1.00	0.75	0.88	0.50	Rejected
**2**	1.00	1.00	1.00	0.75	Accepted
**3**	1.00	0.88	0.94	0.25	Rejected
**4**	1.00	1.00	1.00	0.75	Accepted
**5**	1.00	0.75	0.88	0.50	Rejected
**6**	1.00	1.00	1.00	1.00	Accepted
**7**	1.00	1.00	1.00	0.75	Accepted
**8**	1.00	1.00	1.00	0.75	Accepted
**9**	1.00	1.00	1.00	0.75	Accepted
**10**	1.00	0.88	0.94	1.00	Accepted
**11**	1.00	0.88	0.94	1.00	Accepted
**12**	1.00	1.00	1.00	0.75	Accepted
**13**	1.00	0.88	0.94	0.00	Rejected
**14**	1.00	1.00	1.00	1.00	Accepted
**15**	1.00	0.63	0.82	0.50	Rejected
**16**	1.00	0.88	0.94	0.75	Accepted
**17**	1.00	0.88	0.94	0.25	Rejected
**18**	1.00	0.75	0.88	0.25	Rejected
**19**	1.00	0.75	0.88	0.50	Rejected
**20**	1.00	1.00	1.00	1.00	Accepted

**Fig. 2:**
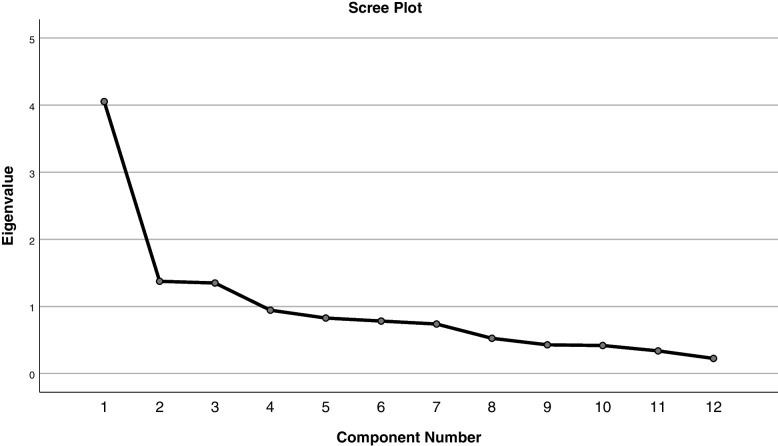
Scree plot of the extracted items of the questionnaire

In EFA, the KMO test was 0.793, indicating the adequacy of the sample size. Bartlett's Sphericity test was also significant (χ2 = 910.163, *P* = 0.000), indicating the suitability of the data for factor analysis. Preliminary EFA analysis showed that none of the items had factor loading lower than 0.40, hence all items were retained. Based on the results of principal components analysis and Varimax rotation and scree plot (Fig. 2), the most suitable three-factor solution was obtained, and these three factors covered 56.48% of the variance. The lowest factor load was 0.525 and the highest was 0.839. Cronbach's alpha coefficient for the final 12 items was 0.791. Table 2 shows the identified factors, distribution of CMAQ items and Cronbach alpha coefficient. The first, second and third factors *—* "perceived interactions in conflict management", "perceived value of conflict management learning" "perceived application of conflict management" *—* includes four, five and three items, respectively.

**Table 2:** Factor loading of items and Cronbach’s alpha coefficient of CMAQ

No	Factor	Items	Factors			Cronbach’s Alpha	Total % of variance
			**1**	**2**	**3**		
1	Perceived interactions in conflict management""	Acquiring conflict management competency improves my ability to effectively communicate with patients, colleagues, and other members of the healthcare team	**0.839**			0.781	56.48
2		Acquiring conflict management competency has helped or will help me be a more effective member of the healthcare team	**0.808**				
3		To become a good physician, I should have the ability to pay attention to various aspects involved in conflict management	**0.792**				
4		Acquiring conflict management competency is as important as having medical knowledge in effective service provision to patients	**0.584**				
5	"Perceived value of learning conflict management"	I see no reason to learn how to manage conflict		**0.750**		0.720	
6		Acquiring conflict management competency is simple and easy		**0.625**			
7		I don't have time to learn conflict management competency		**0.617**			
8		Learning conflict management competency is not applicable in medical profession’ education	0.490	**0.593**			
9		Not having conflict management competency does not detract from my being a physician	0.396	**0.592**			
10	"Perceived application of conflict management"	I find it difficult to use conflict management strategies			**0.813**	0.427	
11		I use conflict management strategies both in life and at work	0.458		**0.601**		
12		It's hard for me to admit that I have trouble using conflict management strategies			**0.525**		

## Discussion

This study aimed at developing and validating medical students’ attitude towards conflict management. The questionnaire was developed based on the steps of the AMEE guide No. 87 [30]. For medical students, this study provided strong evidence of CMAQ’s psychometric properties, adequate internal reliability, and satisfactory factor loading to support its validity. The 12-item CMAQ had acceptable levels of content validity, where all items had a CVI greater than 0.88 and CVR greater than 0.75.

A 12-item self-report questionnaire measuring medical students' attitude towards conflict management. The questionnaire requires participants to imagine being in learning or application of conflict management and asks how much they would perceive the value of having conflict management ability for being good physician. Scores for the total questionnaire and three sub-factors ("Perceived interactions" (4-item), "Perceived value of learning" (5-item) and "Perceived application" (3-item)) are averaged across the 12 items in the total questionnaire and in each factor, to produce values in a range of 0–5. Internal consistency in 250 medical students sample was acceptable (α = 0.79). Although the CMAQ was initially developed as a measure of medical students' attitude, it has also been used to measure physician' and other healthcare practitioners' attitudes towards conflict management.

Exploratory factor analysis led to the identification of a three-factor structure: perceived interactions (attitudes used to establish effective communication and interaction as a member of the treatment team in conflict situations), perceived value of learning (attitudes used to be motivated towards conflict management learning), and perceived application (attitude used to apply conflict management strategies and styles in challenging situations). Depending on items' highest factor loading in aforementioned three factor strictures, items were categorized under their associated high-scored factor structure. Considering that the minimum factor loading of all questionnaire items was higher than 0.40, all items are retained in CMAQ.

An important aspect of the attitude towards conflict management that was identified in the present study was "Perceived interactions in conflict management". The concepts of the five items of this subscale show that acquiring conflict management competency is a necessary qualification for students to play a professional role as a member of the healthcare team in future and to establish effective communication with other team members. Based on Friend et al*.* study, medical and nursing students believed that to succeed in their career, they need to create effective communication in a team and cooperate with each other in clinical settings [37]. Also, Zweibel's study showed that students considered conflicts in healthcare teams to be essential for growth and beneficial, and in their opinion, how to manage conflicts was important. The researchers pointed out the necessity of using communication skills to create a suitable environment for appropriate conflict management [38].

Five items were considered for the "perceived value of learning" subscale. Based on the items of this subscale, students take steps to learn the skills and then apply them in practice based on understanding of the need and value of this competency. According to Barr et al., after completing the conflict management training course, students understood the need for this training and their self-confidence improved in facing conflicts. According to this study, students' positive attitude towards learning conflict management competency and awareness of its importance in their profession means accepting its value and is the first step in the path of skill learning [39]. In line with the present findings, Vandergoot et al*.* emphasized on the existence of a relationship among attitude, motivation to learn, and the transfer of conflict resolution competency in students. The results of their study showed that students with positive motivation and attitude towards conflict management were more motivated to learn conflict management strategies and participating students stated that they will use them in their professional life in future [29].

Based on three items from the subscale of "perceived application", another aspect of the attitude towards conflict management in the present study, if students have a positive attitude towards employing conflict management strategies in their lives and workplaces, they more probably use such strategies in future. Vandergoot et al. highlighted the importance of students’ attitude towards applying conflict management strategies and styles in challenging situations in future [29].

## Strengths and limitations

A strength of the present study was using a systematic framework with the aim of combining the data obtained from qualitative (content analysis of texts and focus group) and quantitative (content and construct validity) methods in the process of developing and validating the questionnaire. Other significant strength of our study is that the items were developed based on information from both representative of medical students and experts, describing their own attitudes towards conflict management. This ensures that attitudes which may not have been previously identified by other researchers can be measured. The CMAQ can be used in combination with observed behavioural and cognitive measures of conflict management to assess all aspects of this competency. It may also have educational implications to assess attitudes towards conflict management along with other behavioural and cognitive information, including those derived from checklist measurement, to enhance the sensitivity and specificity of students' performance; however, the students' performance in clinical practice requires further clinical research to establish.

This study also had several limitations that can be resolved in future studies. CMAQ is a self-report questionnaire, hence it can cause biases due to self-enhancement [40]. CMAQ scores should be checked with other evidence and evaluation sources from multiple perspective. Considering this study, only the opinions of students in one university were collected while information from multicenter and other countries with different cultures can increase the generalizability of using the questionnaire. More studies should be conducted to collect evidence on the validity and reliability of the present findings using developed CMAQ in more communities. Removing some items of the "perceived application" subscale in the psychometric stages reduced the number of questions in this subscale to three. Future studies should also perform confirmatory factor analysis to evaluate CMAQ reliability more strongly.

## Conclusion

This study provided evidence of the validity and reliability of the questionnaire evaluating the medical students’ attitude towards conflict management. For the first time, CMAQ tool including three subscales was developed after exploratory factor analysis. This questionnaire covers three important dimensions of attitude towards conflict management including "perceived interactions", "perceived value of learning" and "perceived application". The present findings showed that participating students considered it valuable and necessary to learn the principles of communication for effective interaction with other team members, and they believe that they need to apply conflict management strategies and styles in challenging situations to succeed in their future careers. Due to lack of evidence on the existence of a specific questionnaire evaluating the attitude towards conflict management. Other future exploratory studies should investigate various aspects of students’ attitudes towards conflict management. This study can be a base for future studies to evaluate the attitude towards conflict management from the perspective of other medical professions such as medical assistants, nurses and other health-science professions.

## Supplementary information

**Additional file 1. **

## Data Availability

The datasets generated and/or analysed during the current study are not publicly available due to the counter’s data sharing policy, but are available from the corresponding author on reasonable request.

## Abbreviations

CMAQ: Conflict Management Attitude Questionnaire
EFA: Exploratory Factor Analysis
CVI: Content Validity Index
CVR: Content Validity Ratio
