# Supplementary material for: Development and validation of conflict management attitude questionnaire for medical students
Source: BMC Med Educ. 2022 Dec 12;22:860. doi: 10.1186/s12909-022-03928-0 (PMC9746217; doi:10.1186/s12909-022-03928-0)
Supplement: Supplementary file 1 — Additional file 1. [file 12909_2022_3928_MOESM1_ESM.docx]

**Additional file 1: Appendix 1. PubMed Search Strategy.**

(((conflict [Title/Abstract]) AND (manag* [Title/Abstract] OR resolution [Title/Abstract])) AND (instrument [Title/Abstract] OR tool [Title/Abstract] OR questionnaire [Title/Abstract] OR Survey [Title/Abstract])) AND ("students, medical" [Title/Abstract] OR "medical student*" [Title/Abstract] OR "Education, Medical" [Title/Abstract] OR "medical education" [Title/Abstract] OR residen* [Title/Abstract] OR "medical Trainees"[Title/Abstract])
